# Use of Instagram and its effect on the mental well-being of university students: A perspective from Pakistan

**DOI:** 10.1371/journal.pgph.0005529

**Published:** 2026-04-29

**Authors:** Ayesha Qamar, Inayat Ali

**Affiliations:** 1 Department of Media and Communication, Fatima Jinnah Women University, Rawalpindi, Pakistan; 2 International Research Institute, Islamic International University, Islamabad, Pakistan; 3 Department of Anthropology, Fatima Jinnah Women University, Rawalpindi, Pakistan; 4 Department of Community Medicine, Shifa Tameer-e-Millat, Islamabad, Pakistan; 5 Department of Social and Cultural Anthropology, University of Vienna, Vienna, Austria; Universiti Kuala Lumpur Royal College of Medicine Perak, MALAYSIA

## Abstract

The global use of social media, particularly Instagram, has considerably increased. At the start of 2024, according to social media platforms, Pakistan recorded 54.38 million social media users aged 18 and older, accounting for 38.9% of the adult population. This rapid digital expansion compelled us to study how Instagram use contributes to social comparison, and its subsequent impact on mental wellbeing among university students. Adopting a quantitative approach, the study conducted online surveys to explore the relationship between Instagram use and mental well-being. A sample of 515 was selected from two well-known universities in Islamabad through convenience sampling. The sample includes 515 male and female students aged between 18 and 25, applying a conditional mediation model (CoMe Model) evaluated by SmartPLS. The findings indicate that increased Instagram use strongly predicts decreased self-esteem (β = -0.661, p < .001), which is linked to higher levels of depression (β = -0.439, p < .001). The indirect effect of Instagram use on depression via self-esteem was significant (β = 0.290, p < .001), while the direct effect became non-significant when self-esteem was included, suggesting full mediation. Importantly, the strength of the mediated pathway varied with levels of upward comparison. The indirect effect was lower among those with high levels of upward comparison (β = 0.116) and stronger among those with low levels (β = 0.201), with the moderated mediation index also reaching significance (β = -0.035, p = .016). These results show that the psychological impact of Instagram use on mental wellbeing is variable and depends on users’ tendency to compare themselves with others.

## Introduction

Globally, the use of social media including Instagram has significantly increased. Instagram ranks third among the most renowned social networking sites [1]. Multiple studies have found a relationship between Instagram usage and mental wellbeing issues [2–5]. Anxiety and depression have been associated with excessive social media usage [6]. Instagram significantly impacted mental well-being, with 56.3% of participants reporting experiences of online hate on the platform, which led them to depression [7]. Depression is a common mental health problem among university students in Pakistan. The high prevalence of depressive symptoms found in this population highlights the importance of examining contributing factors such as social media use [8,9]. In Pakistan, increased Instagram use among young girls substantially correlates with higher body dissatisfaction, poorer mental and physical well-being [10], where mental well-being refers to psychological and emotional health, and physical well-being refers to perceived bodily health and functioning. Likewise, Bint-e-Khalil and Ali (2025) have found that social medial impacts parameters of beauty in women in Azad Jamu and Kashmir (AJK) and affects their mental wellbeing [11]. Individuals with more depressive symptoms compare themselves more upward on Instagram, which further impacts their mental well-being and lead to a vicious circle [12,13]. Servidio and colleagues [14] indicated a significant link between fear of missing out (FoMO), social comparison, and problematic social media usage (PSMU). It was discovered that self-esteem and social comparison sequentially modulated the link between FoMO and PSMU.

Despite the growing body of literature linking Instagram use with mental well-being conditions, it appears there are several important gaps. First, limited empirical evidence exists from low-income countries, particularly Pakistan, where cultural norms, gender expectations, and patterns of social media use may differ substantially from high income countries. Second, previous studies have often examined direct associations between social media use and depression, while not many studies have simultaneously explored the underlying psychological mechanisms, such as self-esteem and upward social comparison, within a single integrative model. Third, research focusing specifically on Instagram—rather than general social media use—among Pakistani university students remains scarce. This study, thus, seeks to address these gaps by examining both mediating and moderating processes in the relationship between Instagram usage and depression within a Pakistani context. It focuses on examining the usage patterns of Instagram among university students in Pakistan and exploring how this social media platform impacts their mental well-being. University students represent a particularly relevant population because they are among the most active users of Instagram, are at a critical developmental stage characterized by identity formation and heightened social comparison, and are at increased risk for depression due to academic, social, and future-related pressures. Additionally, university students are more accessible for early mental health interventions, making them an important group for preventive research.

**Fig 1:**
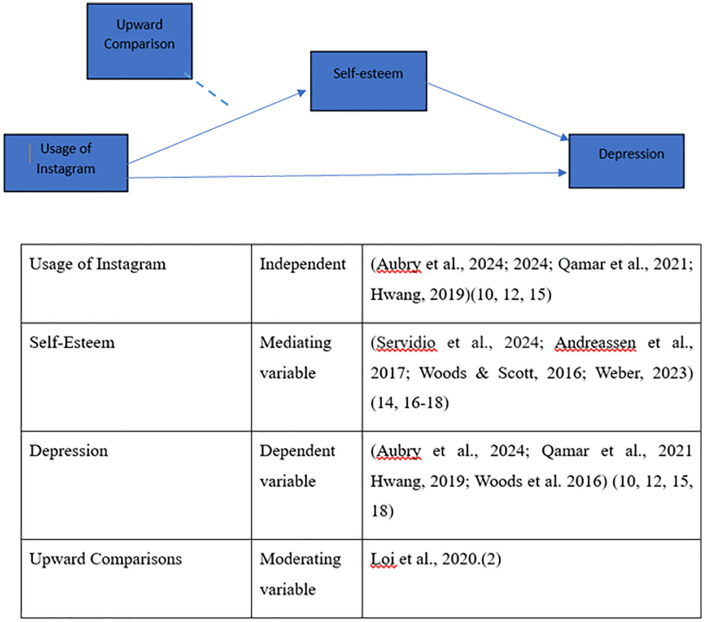
Proposed conceptual model of research [15–18].

In this study, depression is conceptualized as the primary outcome variable, while Instagram usage represents the independent variable (See Fig 1). Self-esteem is defined as an individual’s overall evaluation of self-worth and is examined as a mediating variable, while upward social comparison is defined as the tendency to compare oneself with others perceived as superior and is examined as a moderating variable. Given the high youth population in Pakistan and the increasing popularity of Instagram, it is crucial to understand both potential benefits and risks associated with its use. The study aims to identify specific factors related to Instagram that influence mental well-being, such as levels of social interaction, exposure to visual content, and online social support, while also considering cultural and socio-economic contexts unique to Pakistan. The present study pursues three primary objectives: (1) to investigate the association between Instagram usage and depression among university students in Pakistan; (2) to examine the mediating role of self-esteem in the relationship between Instagram usage and depression; and (3) to assess the moderating role of upward social comparison in the association between Instagram usage and depression among university students. To address these objectives, the study poses the following research questions: RQ1: What is the nature of the relationship between Instagram usage and depression among university students in Pakistan? RQ2: Does self-esteem mediate the relationship between Instagram usage and depression? RQ3: Does upward social comparison moderate the indirect effect of Instagram usage on depression via self-esteem? The central hypothesis (H1) posits that Instagram usage is significantly associated with depression among university students, with self-esteem acting as a mediator and upward social comparison serving as a moderator in this relationship. In the following section, we discuss the conceptual model and the variables used in this study.

### Usage of Instagram

The independent variable includes the usage of instagram (see Fig 1) in which multiple questions were asked from the respondents using 5 item likert sacle of never to very often. I use Instagram daily (UIS). I spend more than 2 hours a day on Instagram (UIS2). I post pictures or videos on Instagram (UIS3). I check others’ posts/stories frequently (UIS4). I compare my life with others based on what they post on Instagram (UIS5).

### Self esteem

Self-esteem is used as a mediating variable in this study, which is measured through a 5-item scale from strongly disagree = 1 to strongly agree = 5: I am satisfied with myself (SE1). I have several good qualities (SE2). I can do things as well as most other people (SE3). I feel that I’m a person of worth, at least on an equal plane with others (SE4). I take a positive attitude toward myself(SE5).

### Depression

Depression (PHQ-9) is measured through a 7-item scale, from not at all to nearly every day = 3. Little interest or pleasure in doing things (D1). Feeling down, depressed, or hopeless (D2). Trouble falling or staying asleep, or sleeping too much (D3). Feeling tired or having little energy (D4). Poor appetite or overeating (D5). Trouble concentrating on things, such as reading or watching TV(D6). Thoughts that you would be better off dead or of hurting yourself (D7).

### Upward comparison

The upward comparison is measured through a 5-item scale from strongly disagree = 1 to strongly agree = 5: I often compare myself with others on Instagram (UC1). Other people on Instagram are doing better than I am (UC2). I feel that others’ lives are more interesting than mine based on their Instagram posts (UC3). I feel less satisfied with my own life after browsing Instagram (UC4).

## Methods and materials

### Study design and setting

This study employed a cross-sectional quantitative research design using an online self-administered survey. Data were analyzed using SmartPLS 4 to examine the effect of Instagram usage on depression, with self-esteem as a mediating variable and upward social comparison as a moderating variable [19]. The study was conducted in Islamabad, the capital city of Pakistan, and data were collected from two large public and private sector universities: Bahria University and International Islamic University Islamabad (IIUI). These universities were selected due to their large and diverse student populations and administrative feasibility for data access, which is consistent with convenience-based survey research in academic settings.

### Target population and participants

The target population comprised undergraduate university students in Pakistan who actively use Instagram. These students were enrolled at Bahria University and IIUI during the data collection period from 20 May 2025 to 20 July 2025.

### Inclusion and exlcusion criteria

The following were inclusion criteria: enrollment as an undergraduate student; age 18 years or older; and active use of Instagram While exclusion criteria included: postgraduate students; students who did not use Instagram; and incomplete survey responses.

Furthermore, a convenience sampling technique was used to select participants. Bahria University has approximately 10,000 students (8,000 undergraduate and 2,000 postgraduate), while IIUI has an enrollment of over 17,000 students, including approximately 7,000 undergraduate students. A total of 515 undergraduate students (260 males and 255 females) completed the survey and were included in the final analysis. The convenience sampling technique was employed due to practical and logistical considerations, including limited access to comprehensive student sampling frames and time constraints associated with multi-institutional data collection. This sampling is commonly used in theory-driven social media and mental health research, particularly when the objective is to examine relationships among psychological constructs rather than to estimate population prevalence. Moreover, undergraduate students constitute a relatively homogeneous group in terms of age, educational context, and social media exposure, making them appropriate for relational and model-testing analyses using PLS-SEM. This approach is consistent with prior empirical studies in the field and is considered suitable for predictive modeling purposes.

### Sample size consideration

The sample size was deemed adequate based on the “10-times rule” commonly applied in partial least squares structural equation modeling (PLS-SEM), which recommends a minimum sample size of ten times the maximum number of structural paths directed at any latent construct in the model. In social science research, the sample size is determined frequently with tables (e.g., Krejcie and Morgan, 1970 [20]) which give the indication that with standard criteria (95 percent level of confidence and 5 percent margin of error) a sample size of about 380–384 people is sufficient to generalize to populations.Given the complexity of the proposed mediation–moderation model, the final sample of 515 respondents exceeded the minimum recommended threshold, ensuring sufficient statistical power for analysis.

### Measurement instruments

All instruments used in this study were standardized and previously validated scales. First, Instagram Usage (Independent Variable) was measured using a 5-item scale assessing the frequency and intensity of Instagram engagement, with responses ranging from “never” (1) to “very often” (5). Second, Depression (Dependent Variable) was measured using the Patient Health Questionnaire-9 (PHQ-9), a widely used and validated scale developed by Kroenke, Spitzer [21] and is commonly applied among university students [22]. Furthermore, responses ranged from “not at all” (0) to “nearly every day” (3), with higher scores reflecting greater depressive symptom severity. Third, while Upward Social Comparison (Moderator) was measured using a 4-item standardized scale adapted from prior studies examining social networking site use and social comparison processes [23,24]. Responses ranged from “strongly disagree” (1) to “strongly agree” (5). Fourth, Self-Esteem (Mediator) was assessed using a 5-item standardized self-esteem scale, with responses ranging from “strongly disagree” (1) to “strongly agree” (5). Higher scores represent higher perceived self-worth.

The validity and reliability of all measurement instruments were evaluated through factor loading, composite reliability, and average variance extracted (AVE) during the measurement model assessment in SmartPLS 4. Items were excluded based on low factor loading, with a retention criterion of 3 established for all variables.

### Data collection procedure and bias control

Data were collected using an online questionnaire distributed via official university communication channels and student social media groups. The survey was administered uniformly across both universities, using the same questionnaire, instructions, and consent procedure to ensure consistency. Participation was voluntary, and no incentives were offered. To minimize selection bias, eligibility criteria were clearly stated at the beginning of the survey, and duplicate responses were restricted through platform settings. Only fully completed questionnaires were retained for analysis.

### Data analysis

**Fig 2:**
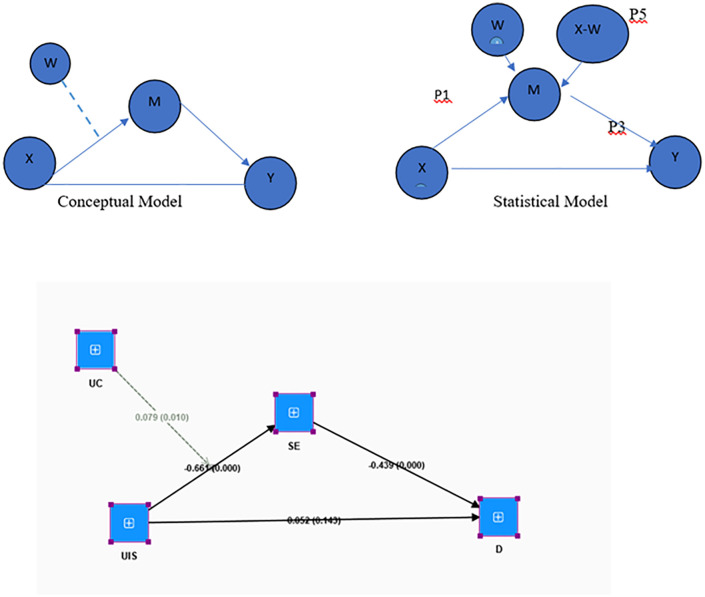
Shows the conceptual, statistical, and structural model, including path coefficients and p-value Instagram and mental health model in SmartPLS.

Data preparation and analysis were conducted using SmartPLS 4 (Fig 2). Preliminary data screening included checking for missing values, response completeness, and outliers, after which incomplete responses were excluded. The analysis followed a two-step PLS-SEM approach: (a) Measurement model assessment, including indicator reliability, internal consistency reliability, convergent validity, and discriminant validity; (b) Structural model assessment, examining direct effects, mediation (self-esteem), and moderation (upward social comparison). Potential confounding variables such as gender and age were controlled for during the analysis. Interaction effects were tested using product indicator approaches, and significance levels were assessed using bootstrapping procedures with 5,000 resamples.

### Ethical statement

The online survey methodology adhered to the Checklist for Reporting Results of Internet E-Surveys (CHERRIES) [25]. The study employed a cross-sectional, closed web-based survey design and received prior institutional review board approval, with electronic informed consent obtained from all participants. Recruitment was limited to eligible undergraduate students through official university channels, and clearly defined inclusion and exclusion criteria were applied. The survey was self-administered, anonymous, and uniformly implemented across both universities. Measures were taken to prevent duplicate entries, and only fully completed questionnaires were included in the analysis. Data handling, statistical analyses, and reporting procedures followed established standards to ensure transparency, data protection, and methodological rigor.

Furthermore, the study adhered to the principles outlined in the Helsinki Declaration and was approved by the relevant institutional review board as per letter number: FJWU/EC/2025/105 on May 19, 2025. Furthermore, we obtained informed consent from all participants to participate and to have their data published. They had full opportunity and willingness to participate in this study or withdraw their participation at any stage. Except their views, no other specifimen were collected from them.

## Results

As mentioned earlier, this study focused on the university students of two universities in Islamabad, Pakistan. The descriptive statistics are shown in Table 1, which mentions that female university students comprised of 255 and male university students are 260 in this study.

**Table 1:** Descriptive statistics of gender.

		Frequency	Valid Percent
Valid	Female	255	49.5
	male	260	50.5
	Total	515	100.0

### Measurement model

The constructs utilized in this study are evaluated through a measurement model executed in Smart PLS4. The assessment of quality criteria initiates with the factor loading assigned to each item within the construct, subsequently followed by an evaluation of both construct reliability and construct validity. Table 2 presents the factor loading of the constructs. All factors exceed 0.50. All items in this study maintained a factor threshold of.50 [26]. Therefore, no items have been removed. The values less than.50 could be removed but in this case, all items have greater than.50, see Table 1.

**Table 2:** Factor loadings.

	D(Depression)	SE(Self Esteem)	UC(upward Comparison)	UIS(Usage of Instagram)
D1	0.810			
D2	0.799			
D3	0.881			
D4	0.868			
D5	0.874			
D6	0.840			
D7	0.848			
SE1		0.835		
SE2		0.874		
SE3		0.554		
SE4		0.799		
SE5		0.823		
UC1			0.840	
UC2			0.942	
UC3			0.593	
UC4			0.941	
UIS1				0.881
UIS2				0.876
UIS3				0.880
UIS4				0.876
UIS5				0.819

### Reliability analysis

As noted by Meadows & Billington (2005), reliability refers to the degree of consistency and stability of the instrument [27]. The primary approaches for assessing reliability are Cronbach’s alpha and composite reliability (CR). Table 3 shows the values of Cronbach’s reliability and Composite Reliability. The Cronbach’s alpha varied from 0.839 to 0.934, while composite reliability ranged from 0.869 to 0.934, indicating the reliability of all constructs under study and exceeding the threshold of 0.70 [28]. Thus, the construct reliability has been established.

**Table 3:** Construct reliability analysis (Cronbach’s alpha and composite reliability).

Variables	Cronbach’s alpha	Composite reliability
D(Depression	0.934	0.934
SE(Self Esteem)	0.839	0.869
UC(upward Comparison)	0.855	0.908
UIS(usage of Instagram)	0.917	0.918

### Convergent validity and discriminant validity

Convergent validity refers to the extent to which various methods of measuring the same concept yield consistent results. When the average variancer extracted value (greater than or equal to 0.50) convergent validity was established [29]. The current study demonstrated that all constructs have AVE values exceeding 0.50, thereby confirming the establishment of convergent validity. Table 4 presents the AVE value for each construct.

**Table 4:** Construct Convergent validity(AVE).

	Average variance extracted (AVE)
D(Depression	0.716
SE(Self Esteem)	0.616
UC(upward Comparison)	0.708
UIS(usage of Instagram)	0.752

### Discriminant validity

The extent to which measurement concepts are differentiated. The concept suggests that when two or more ideas are distinct, the valid measures for each should not exhibit a high degree of correlation [30]. The researcher used the Fornell-Larker criteria for validating discriminant validity.

#### Fornell Larker criterion.

Fornell and Larcker [29] state that criterion discriminant validity is confirmed when the square root of the Average Variance Extracted (AVE) for a construct exceeds its correlation with all other constructs. In this study, the square root of the Average Variance Extracted (AVE) for a construct exceeds its correlation with other constructs—Table 5. Therefore, discriminant validity is affirmed.

**Table 5:** Average variance extracted.

	D	SE	UC	UIS
D	***0.846***			
SE	-0.498	***0.785***		
UC	0.726	-0.639	***0.841***	
UIS	0.329	-0.625	0.358	***0.867***

Bold and italics represent the square root of AVE of all constructs used in this study(Depression, Self Esteem, upward Comparison and Usage of Instagram).

### Structural model

#### Conditional mediation analysis.

We verified the complex model to check the mediation and see whether the moderator is changing the indirect effect. The usage of Instagram leads to depression through self-esteem and upward comparison, which negatively influences the relationship between usage of Instagram and self-esteem [31]. Conditional mediation is used when a model has one mediator(self esteem) and one moderator(upward comparison). An index term analysis is conducted when the moderator is continuous, and CoMe aims to assess the rate of change or relationship.

We analyzed the relationship between Instagram usage and depression, focusing on the role of self-esteem as a mediator and the moderating effect of upward comparison. Researchers examine the influence of the moderator (UC) on the indirect pathway within a more intricate model. In the Conditional mediation analysis (CoMe)model, the independent variable (UIS - Usage of Instagram) influences the outcome variable Y (Depression D) through one mediator M (SE - self-esteem), and the relationship is conditioned by one moderator W, upward comparison UC [31]. The p-value in Table 6 indicates that all values are less.05 at 95% confidence interval. Hence, *H1* of this study is accepted.

**Table 6:** Moderated mediation.

	Original sample (O)	Sample mean (M)	Standard deviation (STDEV)	T statistics (O/STDEV)	P values
UC - > SE - > D	0.229	0.233	0.058	3.953	0.00
UIS - > SE - > D	0.29	0.289	0.069	4.178	0.00
UC x UIS - > SE - > D	-0.035	-0.034	0.016	2.138	0.016

In the context of conditional mediation analysis, index term analysis is employed. The indices of P2 and P5 can be directly obtained from SmartPLS, as illustrated in Fig 1. The findings indicate that P2*P5 (or UC*UIS - > SE - > D) is statistically significant (p < .05). Given that the index “P2*P5 of UC is significant (β = -0.035), it can be concluded that the mediated effect of UIS on D through ES is negatively influenced by the moderator UC.

Index =-0.035 (.079 (UC*UIS->SE)* -0.439(SE->D)).

#### Reporting moderated mediation.

Table 7 explains the direct relationship between constructs and answers RQ1. A notable negative correlation was identified between Instagram usage and self-esteem (β = -0.661, t = 5.091, p < .001), suggesting that increased use of Instagram is linked to diminished self-esteem. Self-esteem was found to be a significant predictor of depression (β = -0.439, t = 6.302, p < .001), indicating that lower self-esteem correlates with increased levels of depression. Instagram usage demonstrated a notable direct effect on depression (β = 0.343, t = 5.349, p < .001), indicating a positive association with depression that persists beyond the indirect impact.

**Table 7:** Direct relationship.

	Original sample (O)	T statistics	P values
Self-esteem - > Depression	-0.439	6.302	0.000
Usage of Instagram - > Depression	0.343	5.349	0.000
Usage of Instagram - > Self-esteem	-0.661	5.091	0.000
Upward comparison x Usage of Instagram - > Self-esteem	0.079	2.281	0.011

*A T-statistic value greater than 1.645 means it is significant at.005.

The interaction between upward comparison and Instagram usage on self-esteem was found to be significant (β = 0.079, t = 2.281, p = .011), which suggests that the impact of Instagram usage on self-esteem is influenced by the degree of upward comparison. This indicates the existence of a moderation effect.

Table 8 answered RQ2. The indirect effect of Instagram on depression through self-esteem is significant at the.000 level. The analysis examined whether self-esteem serves as a mediator in the relationship between Instagram use and depression, assessing the significance of this indirect pathway. The findings indicated a significant indirect effect of Instagram use on depression through self-esteem (indirect effect = 0.290, t = 4.178, p < .001). This suggests that Instagram use contributes to increased depression, in part by diminishing self-esteem, which subsequently leads to higher levels of depression. Conversely, the direct effect of Instagram use on depression, after considering the influence of self-esteem, was not significant (direct effect = 0.052, t = 1.069, p = 0.143). The analysis confirmed that the indirect effect (Instagram ➝ self-esteem ➝ depression) is significant (p < .001), while the direct effect (Instagram ➝ depression) is not significant (p = 0.143) when self-esteem is included in the model. Thus, the relationship between Instagram use and depression is fully mediated by self-esteem, and the model is successfully established and confirmed through SmartPLS.

**Table 8:** Moderated indirect relationship.

	direct effect	indirect effect
Usage of Instagram - > self-esteem - > depression	.052 (1.069)	.290 (4.178)
p value	0.143	0.000

The above-mentioned Table 9 indicates that the indirect path of usage of Instagram (UIS) leads to depression (D) through the mediator (SE), demonstrating a low value at a high level of the moderator (UC) =.0116. Conversely, the value of the indirect effect increases at a lower level of the moderator (UC) =.201. Although it is significant at all levels of moderation. The table indicates that Instagram (UIS) indirectly influences depression (D) through self-esteem (SE), which is impacted by upward comparison (UC). Instagram has a slight indirect influence on depression (.0116) when people compare themselves to others (high UC). The effect is larger (.201) when individuals make fewer comparisons (low UC). If people do not compare themselves to others, Instagram use might diminish self-esteem and cause depression. Although this indirect impact is present at all comparison levels, its intensity depends on the extent of the comparison.

**Table 9:** Conditional indirect effect at different levels of the moderator (UC).

	Original sample (O)	Sample mean (M)	Standard deviation (STDEV)	T statistics (O/STDEV)	P values
UIS - > SE - > D conditional on UC at +1 SD	0.116	0.118	0.034	3.452	0.000
UIS - > SE - > D conditional on UC at -1 SD	0.201	0.203	0.036	5.527	0.000
UIS - > SE - > D conditional on UC at Mean	0.159	0.16	0.027	5.905	0.000

## Discussion

This study examined a moderated mediation model to analyze the relationship between Instagram use (UIS) and depression (D), with self-esteem (SE) serving as a mediator and upward social comparison (UC) acting as a moderator. The findings indicate a nuanced relationship among these variables, offering valuable insights into the ways social media platforms such as Instagram can influence users’ mental wellbeing, especially concerning self-perceptions and tendencies toward comparison.

Our research demonstrates that self-esteem plays a significant mediating role in the relationship between Instagram usage and depression. Increased usage of Instagram correlated with decreased self-esteem which subsequently predicted elevated levels of depression.This aligns with research indicating that regular interaction with social media platforms, particularly those focused on images such as Instagram, may have detrimental effects on self-esteem [32]. The indirect relationship between Instagram usage and depression via self-esteem was found to be statistically significant.In contrast, the direct effect was rendered non-significant upon the inclusion of the mediator.This pattern indicates complete mediation, which suggests that Instagram’s effect on depression is primarily mediated by its influence on users’ self-esteem.

The analysis revealed that upward comparison significantly interacted with Instagram usage in predicting self-esteem thereby confirming the existence of a moderation effect. The conditional indirect effect of Instagram use on depression through self-esteem was found to be weaker at high levels of upward comparison and stronger at low levels of upward comparison while remaining significant across all levels. The index of moderated mediation was significant, indicating that the strength of the indirect path fluctuates according to the levels of upward comparison.

This finding may seem paradoxical initially; one might anticipate that individuals who often engage in upward comparison would experience a greater impact from Instagram use regarding self-esteem and depression. Our findings indicate a contrary perspective: the influence of Instagram usage on self-esteem, and consequently on depression, is more significant when upward comparison is minimal. A potential reason is that individuals strongly inclined to compare themselves to others may experience persistently low self-esteem, indicating that Instagram use does not significantly exacerbate their self-assessments. In their case, depression might be influenced by additional psychological factors not addressed in this study, including envy [33], anxiety about missing out [34], or feelings of social isolation [35].

On the other hand, individuals who engage less in upward comparison may not frequently evaluate their self-worth in everyday situations. However, when individuals engage extensively with Instagram, a platform characterized by idealized images, they may encounter a significant decline in self-esteem due to their lack of familiarity with such social comparisons. This abrupt change may clarify why the indirect impact of Instagram usage on depression is more pronounced among these individuals. The findings are consistent with the dual-pathway model of social media effects put forth [36], indicating that social comparison and self-esteem collaboratively influence the mental health associated with social media usage.

The notable indirect effect observed across all levels of upward comparison suggests that self-esteem consistently influences the relationship between Instagram use and depression. Nonetheless, the influence of this role fluctuates, underscoring the necessity of accounting for individual variations in social comparison tendencies when examining the effects of social media. Our findings align with previous research indicating that social media can adversely affect well-being by reducing self-esteem [23,37].

Some studies have reported varying outcomes. Verduyn, Ybarra [38] posited that passive social media use, characterized by scrolling without interaction, results in an adverse effect due to upward comparison, in contrast to active use, which does not have the same effect. Our study did not distinguish between passive and active use, which could be considered a limitation. Furthermore, Tandoc Jr, Ferrucci [39] indicated that the use of Facebook is a predictor of depression, mainly through the mechanisms of social comparison and rumination. This reinforces the notion that the psychological effects of social media platforms are contingent upon context and shaped by individual traits and behaviors.

An alternative interpretation of our findings may be found in the concept of social comparison orientation [40] which denotes a predisposition to evaluate oneself in relation to others. Individuals with a strong inclination towards social comparison may experience a consistent impact from upward comparisons, while for others, Instagram could catalyze infrequent comparisons, resulting in heightened emotional reactions. It has been found that individuals evaluate their own worth by comparing themselves to others [41,42]. In the context of Instagram, such comparisons often involve idealized portrayals of lifestyles, appearances, and accomplishments, commonly leading to decreased self-esteem. This research advances the theoretical framework by illustrating that the impact of these social comparisons on self-esteem and depression is moderated by an individual’s inherent tendency to engage in upward comparisons. This research comprised the usage of social media among young university students of sample size of 515 established the external validity that exceeds the statistical threshold (n = 384)recommended by Krejcie and Morgan (1970). Consequently, the findings possess sufficient statistical power to be generalized to the broader population.

## Implications

The findings have significant implications for mental wellbeing interventions and digital literacy programmes. Educating users, especially young adults, about the potential harmful effects of upward comparison and promoting mindful engagement with social media content can help lessen its negative impact on mental wellbeing. Psychological strategies such as mindfulness and self-compassion can be effectively taught to reduce social media-related comparison and boost self-esteem. In universities, Students’ Counselling Centre can use these findings, and different seminars on mental wellbeing can be organised. Additionally, platform designers might explore features that minimize comparison drivers, such as concealing like counts or encouraging a variety of authentic content. It is essential for practitioners engaging with young people to evaluate their social media usage and their inclination towards social comparison as a component of comprehensive mental wellbeing care.

## Limitations and future research

This study has certain limitations. The cross-sectional nature of the data limits the ability to establish causality. Future research should employ longitudinal or experimental designs to confirm the direction of effects. Additionally, distinguishing between different types of Instagram use (such as active versus passive) and expanding the model to include additional mediators (such as envy, FoMO, and loneliness) may offer a more comprehensive understanding of the links between social media engagement and mental health outcomes. The generalization of the findings is constrained by the use of convenience sampling. Unlike probability sampling, which ensures every member of the population has an equal chance of selection, convenience sampling relies on participants who are readily accessible. Consequently, the sample may suffer from selection bias. Furthermore, since depression is influenced by multiple confounding factors such as gender, age, and family-related factors, it is suggested that future studies should include these variables as confounders in the model.

## Conclusion

This study provides compelling evidence demonstrating that self-esteem functions as a full mediator in the relationship between Instagram usage and depression. By highlighting the complex interplay between online behaviors and psychological outcomes, our findings demonstrate that this mediation process appears to be significantly influenced by upward social comparison. These findings highlight the importance of considering individual differences in social comparison tendencies when assessing the psychological effects associated with social media platforms. As Instagram plays a prominent role in shaping self-perceptions and emotional wellbeing, it is essential for researchers and mental health practitioners to thoroughly understand these intricate pathways. Such insights can inform targeted interventions and guide the development of strategies aimed at mitigating the adverse effects of social media engagement on mental wellbeing, ultimately promoting healthier online interactions and psychological resilience.

## Supporting information

S1 Data Data file. (XLSX)
